# Hepatocellularcarcinoma: current imaging techniques

**DOI:** 10.1186/1470-7330-15-S1-P4

**Published:** 2015-10-02

**Authors:** S De Luca, E Casalini Vañek, A Vazquez, M Wirtz, F Troncoso, E Eyheremendy

**Affiliations:** 1 Hospital Aleman, Buenos Aires, Argentina

## Learning objectives

Describe the use of current imaging techniques to reach a correct diagnosis of hepatocellular carcinoma.

Emphasise the knowledge of complementary tools for the evaluation of early response to treatment tumour.

## Content organisation

Our cases will be presented in a pictorial essay mode.

It is important to consider:

The radiologic diagnosis of HCC can be made by either CT or MRI methods.

Typically, HCC enhances during the arterial phase due to the presence of an intense arterial blood supply of the hepatic arteries.

TC dual Energy: Dual-energy CT (DECT) is an innovative imaging technique that operates on the basic principle of application of two distinct energy settings that make the transition from CT attenuation–based imaging to material-specific or spectral imaging. DECT can also aid in evaluation of response to therapy and detection of oncology-related disorders.

MDCT perfusion: non-invasive method of quantification of tumour blood supply, related to the formation of new arterial structures (neoangiogenesis), which are essential for tumour growth.

## Conclusion

Dual energy and perfusion CT are innovative techniques to detect suspicious lesions of hepatocarcinoma.

Dual Energy CT also helps to reduce radiation exposure by using low dose imaging protocols without affecting diagnostic purpose.

The Perfusion provides information on the neovascularization of an injury (predicting malignancy) and assesses early therapeutic response (before displaying morphological changes).

